# Water-Dispersible and Biocompatible Polymer-Based Organic Upconversion Nanoparticles for Transdermal Delivery

**DOI:** 10.34133/bmr.0106

**Published:** 2024-11-19

**Authors:** Hye Eun Choi, Jeong-Min Park, Woo Yeup Jeong, Su Bin Lee, Jae-Hyuk Kim, Ki Su Kim

**Affiliations:** ^1^ School of Chemical Engineering and Institute for Advanced Organic Materials, Pusan National University, Busan 46241, Republic of Korea.; ^2^ Department of Civil and Environmental Engineering, Pusan National University, Busan 46241, Republic of Korea.; ^3^ Department of Organic Materials Science and Engineering, Pusan National University, Busan 46241, Republic of Korea.

## Abstract

Photomedicine, which utilizes light for therapeutic purposes, has several hurdles such as limited tissue penetration for short-wavelength light and inadequate deep tissue efficacy for long-wavelength light. Photon energy upconversion (UC) reveals promise in photomedicine because it enables the conversion of lower-energy photons into higher-energy photon. Lanthanide (Ln)-based inorganic UC system has been extensively studied but faces challenges, including high excitation laser power density, intrinsically subpar UC quantum efficiency, and potential biotoxicity. Recently, an organic-based triplet–triplet annihilation UC (TTA-UC) system has emerged as a novel UC system due to its prolonged emission lifetime upon low power laser excitation and exceptional UC quantum yield. In this study, we developed water-dispersible hyaluronic acid (HA)-conjugated polycaprolactone (PCL) nanoparticles loaded with TTA-UC chromophores (HA-PCL/UC NPs), which allow deeper tissue penetration by converting red light (635 nm) into blue light (470 nm) for noninvasive transdermal delivery. HA-PCL/UC NPs demonstrated a 1.6% high quantum yield in distilled water, improved cellular imaging in HeLa cells, and effectively penetrated the deep tissue of porcine skin, showing upconverted blue light. Our strategy holds significant potential as a next-generation noninvasive photomedicine platform for bioimaging, photo-triggered drug delivery, and photodynamic therapy, ultimately advancing targeted and effective therapeutic interventions.

## Introduction

Photomedicine, an emerging therapeutic approach that utilizes light for diverse applications, offers inherent therapeutic effects and facilitates other therapeutic effects [1]. This approach harnesses the unique characteristics of light based on wavelength, leading to versatile applications. Among various wavelengths, near-infrared (NIR) light is commonly used in thermal therapy due to its heat properties and ability to induce photothermal effects [2,3]. Conversely, ultraviolet (UV) light serves as the stimulator, promoting antibacterial effects or activating other pharmacological effects owing to its high energy [4]. Despite their potential, photomedicine encounters challenges because of the properties of light. Long-wavelength light is effective for reaching and acting within deep tissue but may lack sufficient energy to activate adequate reactions. In contrast, short-wavelength light possesses higher energy and is capable of triggering chemical reactions but holds boundaries about penetration restrictions when exposed externally to the body.

In the pursuit of surmounting these constraints, photon energy upconversion nanoparticles (UC NPs) have shown great promise in various photomedicine fields. UC involves the conversion of lower-energy photons into a single photon with higher energy, possessing the advantages of a broad hypochromatic shift, sharply defined emission peaks, extended luminescence shelf-life, exceptional photostability, and improved penetration into biological tissues [5,6]. Based on these attributes, UC has been developed in photomedicine for applications such as bioimaging [7], drug delivery [8], photothermal therapy [9], photodynamic therapy [10], immunotherapy [11], neuromodulation [12], and photochemical tissue bonding [13]. In the UC landscape, particular attention has been directed toward inorganic (rare earth ion)-based UC NP system. Inorganic lanthanide (Ln)-doped UC NPs exhibit minimal photodegradation, significant anti-Stokes shifts, manageable emission spectra, ease of multifunctional integration, size manipulation, phase adjustability, and enduring photostability. However, inorganic UC NPs encounter various hurdles, including the requirement for a relatively intense excitation power density (>10^3^ to 10^5^ mW cm^−2^), the intrinsic subpar UC quantum yield (<0.001%), and potential toxicity associated with Ln ions when situated at the target site [14].

To overcome these problems, an organic-based triplet–triplet annihilation UC (TTA-UC) system has been developed as an emerging alternative [15]. TTA-UC system includes a photosensitizer and an acceptor, where photon UC occurs through excitation of the photosensitizer to its singlet excited state, followed by intersystem crossing (ISC), triplet energy transfer to the acceptor, triplet fusion between triplet acceptors, and production of a singlet excited acceptor, finally resulting in higher-energy photon emission [16–18]. Notably, TTA-UC demonstrates a prolonged emission lifetime (up to several tens or hundreds microseconds) compared to the original fluorescence lifetime (nanoseconds) upon excitation by a low-power laser (<10^2^ mW cm^−2^ or ambient sunlight), remarkable quantum efficiency (>1 to 40%), an intense absorption coefficient of the sensitizer, and ease of tunable excitation and emission wavelengths. A great deal of research has investigated TTA-UC configuration based on organic solvents, such as toluene and chloroform, which constrain their applicability for in vivo applications [19]. Clearly, TTA-UC faces several challenges for biological applications because it is advantageous to be adaptable to aqueous conditions. An aqueous environment engenders poor solubility of hydrophobic photosensitizers and acceptors while concurrently elevating the risk of triplet state quenching by dissolved molecular oxygen. Emerging strategies encompassing the development of silica or polymer coatings have been developed to address these problems and serve as carriers for hydrophobic TTA-UC chromophores with or without oxygen scavengers [20–23].

On the other hand, in the realm of medical interventions involving photomedicine, the intravenous injection of photosensitizers and photothermal agents remains the predominant mode of administration. However, this method is burdened by several constraints, for example, insufficient drug accumulation within cutaneous lesions, dispersion of drugs across multiple organs, and undesirable drug leakage. Although intravenous administration permits the delivery of substantial quantities of photomedicine agents, this approach presents the accompanying high risk of systemic toxicity. A compelling alternative has emerged in the form of transdermal drug delivery system (TDDS), which holds promise in surmounting these challenges. A TDDS allows direct targeting of photomedical agents to precisely localized cutaneous lesions while ensuring heightened patient adherence and comfort [24]. Notably noninvasive and devoid of discomfort, the TDDS facilitates the controlled and deliberate systemic circulation of drugs through skin tissue [25]. In the field of photomedicine, the use of light to deliver energy noninvasively to the body presents a promising avenue. Leveraging this capability, TDDS has emerged as a viable method, potentially synergizing with light-based therapies to enhance their therapeutic efficacy. Wang et al. [26] discussed a novel TDDS for targeted photodynamic therapy against bacterial skin infections, using a chlorin e6 (Ce6)-conjugated catalase and a fluorinated low molecular weight polyethyleneimine formulation to achieve effective in vivo antibacterial results. However, the presence of a formidable stratum corneum, the outermost skin barrier of considerable thickness, poses an obstacle to the delivery of external substances. To address this issue, hyaluronic acid (HA), a linear biodegradable natural polymer that is widely recognized for its biocompatibility, has been explored as a potential solution. Among the various advantages of HA, we focused on its potential to enhance transdermal delivery efficiency, as demonstrated in our previous study [27]. Despite its high hydrophilicity and large molecular size, HA can infiltrate the stratum corneum and reach cells in the dermis, enhancing the transdermal delivery efficiency [28]. The precise mechanism underlying the transdermal delivery of HA is not yet fully understood, but the proposed explanations include hydration-induced disturbance of the keratin structure and infiltration of HA through intercellular lipids, facilitated by its amphiphilic nature.

**Fig. 1.**
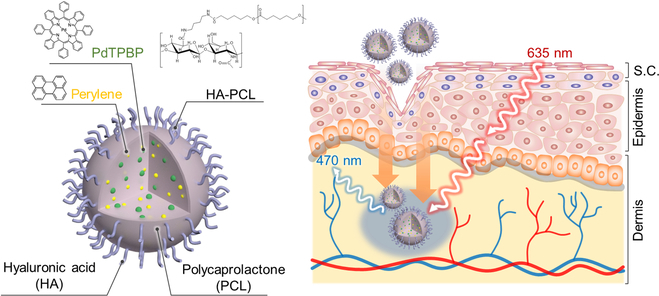
Schematic illustration of water-dispersible and red-to-blue upconverting HA-PCL/UC NPs for transdermal delivery system.

Herein, we propose a strategy for overcoming the challenges associated with photomedicine and TDDS by developing water-dispersible red-to-blue upconverting HA-conjugated polycaprolactone (PCL) nanoparticles (HA-PCL/UC NPs) (Fig. 1). Notably, the TTA-UC property was imparted to the PCL NPs by simply mixing a representative UC chromophore pair, palladium (II) meso-tetraphenyl-tetrabenzo-porphyrin (PdTPBP), as a sensitizer and perylene as an acceptor, with commercially available PCL during the NP preparation step. This method eliminates the need for high-temperature sintering process, which is essential for the synthesis of inorganic UC NPs. The use of PCL as a widely used polyester material in biomedical applications allows for degradation via hydrolysis under physiological conditions [29], facilitating the loading of hydrophobic TTA-UC chromophores and reducing susceptibility to skin barrier function. Furthermore, HA-based polymeric UC NPs promote noninvasive transdermal delivery into deep skin tissues. This combination enables the conversion of red light into blue light, allowing for deeper penetration into the skin tissue. We investigated strategies for synthesizing HA-PCL conjugates and HA-PCL/UC NPs and examined the particle properties, optical analysis, in vitro analysis, and transdermal efficiency using porcine skin for comparing PCL/UC NPs.

## Materials and Methods

### Materials

Materials and solvents were acquired from commercial suppliers and used without further purification. Sodium hyaluronate (HA; 100 kDa) was purchased from SNVIA Co. Ltd. (Busan, Korea). 1-Ethyl-3-(3-dimethylaminopropyl) carbodiimide (EDC; >98%), *N*-hydroxysuccinimide (NHS; 98%), 1,4-diaminobutane (DAB), perylene, *N*,*N*-dicyclohexylcarbodiimide (DCC), and palladium(II) acetate were purchased from Tokyo Chemical Industry Co. Ltd. (Tokyo, Japan). PCL (14,000 kDa), polyvinyl alcohol (PVA), sodium hydroxide, phenylacetic acid, tetrahydrofuran (THF), dichloromethane (DCM), and phthalimide were purchased from Sigma-Aldrich (St. Louis, MO). Dulbecco’s modified Eagle’s medium (DMEM), fetal bovine serum (FBS), penicillin, and phosphate-buffered saline (PBS) were purchased from Invitrogen (Carlsbad, CA). MAX-View Live/Dead Cell Staining Kit was purchased from Biomax (Guri, South Korea).

### Synthesis of HA-PCL conjugates

HA-PCL is synthesized by conjugating the carboxyl groups of HA and PCL with a diamine linker. Initially, a DAB-grafted HA conjugate (HA-DAB) was prepared as previously described in the literature [30]. Briefly, 100 mg of HA was dissolved in 50% (v/v) ethanol/water. After complete dissolution, DAB (40 molar ratios of HA repeating units) was added to the HA solution along with EDC/NHS (2 molar ratios of HA repeating units). Then, the well-mixed solution was kept at pH 5.5 for 24 h. Subsequently, HA-DAB was purified by 3 d of dialysis (molecular weight cutoff = 10 kDa) and obtained by lyophilized for 3 d. After that, to synthesize HA-PCL conjugates, HA-DAB and PCL (4 molar ratios of the amino group of the HA-DAB conjugate) were dissolved in THF. DCC/NHS (10 molar ratios of the amino group of HA-DAB) was then added and stirred for 24 h. Finally, the mixture was dialyzed against deionized water (DW) for 3 d and lyophilized for 3 d. The substitution ratio of HA-PCL was characterized using ^1^H nuclear magnetic resonance spectroscopy (^1^H-NMR, AVANCE NEO 500, Bruker BioSpin, Rheinstetten, Germany). CDCl_3_ was used as a solvent, and tetramethylsilane was the standard for which δ = 0.00 ppm. Fourier transform infrared spectroscopy (FTIR) was performed to detect the information of chemical bonds (Nicolet iS50, Thermo Scientific, Madison, WI).

### Preparation and characterization of HA-PCL/UC NPs

HA-PCL/UC NPs were fabricated using the oil-in-water (O/W) emulsion method with a tip homogenizer. PdTPBP was synthesized as previously reported [31]. To encapsulate the UC chromophore into HA-PCL NPs, PdTPBP (9.03 × 10^−4^ M) and perylene (2.37 × 10^−2^ M) were dissolved in 4 ml of HA-PCL solution (DCM, 1 w/v%). The solution was added to 20 ml of 2% (w/v) PVA aqueous phase solution, and the mixture was subsequently emulsified using a tip homogenizer (VC 750, Sonics and Materials Inc., Newton, CT) at 200 W of energy output for 20 min in an ice bath. After that, the prepared O/W emulsion was vigorously stirred overnight to evaporate the organic solvent. The fabricated HA-PCL/UC NPs were washed 3 times by ultracentrifugation at 11,000 rpm for 20 min. After centrifugation, the precipitated NPs were dispersed in 4 ml of DI water. The UC chromophores encapsulated in PCL NPs were prepared according to the same procedure described above.

The morphologies of the samples were visualized using a 120 kV transmission electron microscope (TEM, Hitachi, Japan). The size distributions and zeta potentials of the different samples were analyzed using dynamic light scattering (DLS; Zetasizer Nano ZS90, Malvern, Worcestershire, UK). To evaluate particle stability, particles at a concentration of 10 mg ml^−1^ were individually dispersed in DW and PBS. The hydrodynamic diameters of these particles were monitored over a 7-d period using DLS.

### Optical analysis

The absorption and emission spectra, phosphorescence, and UC emission lifetimes of the samples (UC chromophores in DCM and 120 mg ml^−1^ of HA-PCL/UC NP aqueous suspension) were obtained using a spectrofluorometer with a 635 nm commercial diode laser (FS5-TCSPC, Edinburgh Instruments, Edinburgh, UK). The power density of the incident laser was calculated by measuring the incident laser intensity using a power meter (834-R, Newport Corporation, Irvine, CA), and the scattered laser light was removed using a 632 nm notch filter. The UC quantum yield (*Φ*_*UC*_) of HA-PCL/UC NPs was calculated via Eq. 1.

$$\Phi_{\text{UC}}=\Phi_{\text{std}}\left(\frac{A_{\text{std}}}{A_{\text{UC}}}\right)\left(\frac{I_{\text{UC}}}{I_{\text{std}}}\right){\left(\frac{\eta_{\text{UC}}}{\eta_{\text{std}}}\right)}^{2}$$ (1)

where *Φ*, *A*, *I*, and *η* denote the quantum yield, absorbance at 635 nm, integrated emission intensity, and refractive index of the medium, respectively. The subscript “UC” and “std” refer to the HA-PCL/UC NPs and the reference standard (HA-PCL/PdTPBP NPs, *Φ*_*std*_ = 7%), respectively.

### Cell lines

Human cervical carcinoma HeLa cells and mouse fibroblast L929 cells were purchased from the Korean Cell Line Bank (Seoul, Korea) and cultured in DMEM containing 10% FBS and 1% penicillin/streptomycin under a humidified 5% CO_2_ atmosphere at 37 °C.

### Cell viability analysis

The viability of HA-PCL/UC and PCL/UC NPs was assessed in HeLa and L929 cells using the CCK-8 assay. Cells were cultured in 96-well plates at 1 × 10^4^ cells per well. After a 24-h incubation, NPs at various concentrations of 0, 5, 50, 100, 200, and 500 μg ml^−1^ were added to each well and further incubated for 24 h. Then, the medium was replaced with fresh DMEM and 10 μl of CCK-8 kit solution. The CCK absorbance was recorded at 450 nm with the assistance of a microplate spectrophotometer. Additional Live/Dead assays were conducted with HeLa and L929 cells. Cells were seeded at a density of 0.5 × 10^5^ cells per well in 24-well plates. After 24 h of culture, cells were incubated with NP suspensions at a concentration of 500 μg ml^−1^ for 4 h. Then, the cells were washed 3 times with fresh PBS and incubated with Live/Dead assay solution for 10 min. The stained cells were observed under a fluorescence microscope (EVOS M5000, Thermo Fisher Scientific, Waltham, MA).

### In vitro fluorescence and UC imaging based on cellular uptake assessment

To assess the degree of cellular uptake, PCL/perylene, PCL/UC, HA-PCL/PdTPBP, and HA-PCL/UC NPs were incubated in HeLa cells. HeLa cells were seeded on coverslips in 12-well plates at a density of 1 × 10^5^ cells per well and incubated with 500 μg ml^−1^ of NPs. Afterward, the cells were washed 3 times with PBS and fixed with 4% paraformaldehyde. The fixed cells were washed twice with fresh PBS and mounted. Finally, the cells were observed under a confocal laser scanning microscope (CLSM; ZEISS LSM 800, Oberkochen, Germany).

### Skin penetration evaluation

To examine the depth profile of the infiltrated NPs in the skin, PCL/UC and HA-PCL/UC NPs were topically applied to porcine skin, purchased from local slaughterhouse. A 200-μl aliquot of NPs (500 μg ml^−1^) diluted in PBS was prepared and applied to porcine skin. Then, at the predetermined times of 2, 4, 8, 12, and 24 h, skin tissues were collected into an area of 1 cm × 0.5 cm and fixed on the slide glass with the cross-section facing up. The prepared samples were analyzed using CLSM to confirm whether blue light was generated in the skin tissue when irradiated with 405- and 640-nm lasers. The obtained images were analyzed for the intensity of blue light according to depth using ImageJ (National Institutes of Health, Bethesda, MD), and the transdermal delivery efficiency was compared between each sample.

### Statistical analysis

All data are expressed as means ± standard deviation (SD) from at least 7 independent experiments. Statistical analysis was conducted using 2-way analysis of variance (ANOVA) with Bonferroni post hoc via GraphPad Prism 5.0 (GraphPad Software Inc., La Jolla, CA). The values of *P < 0.05, **P < 0.01, and ***P < 0.001 were considered significant.

## Results

### Preparation and characterization of HA-PCL/UC NPs

**Fig. 2.**
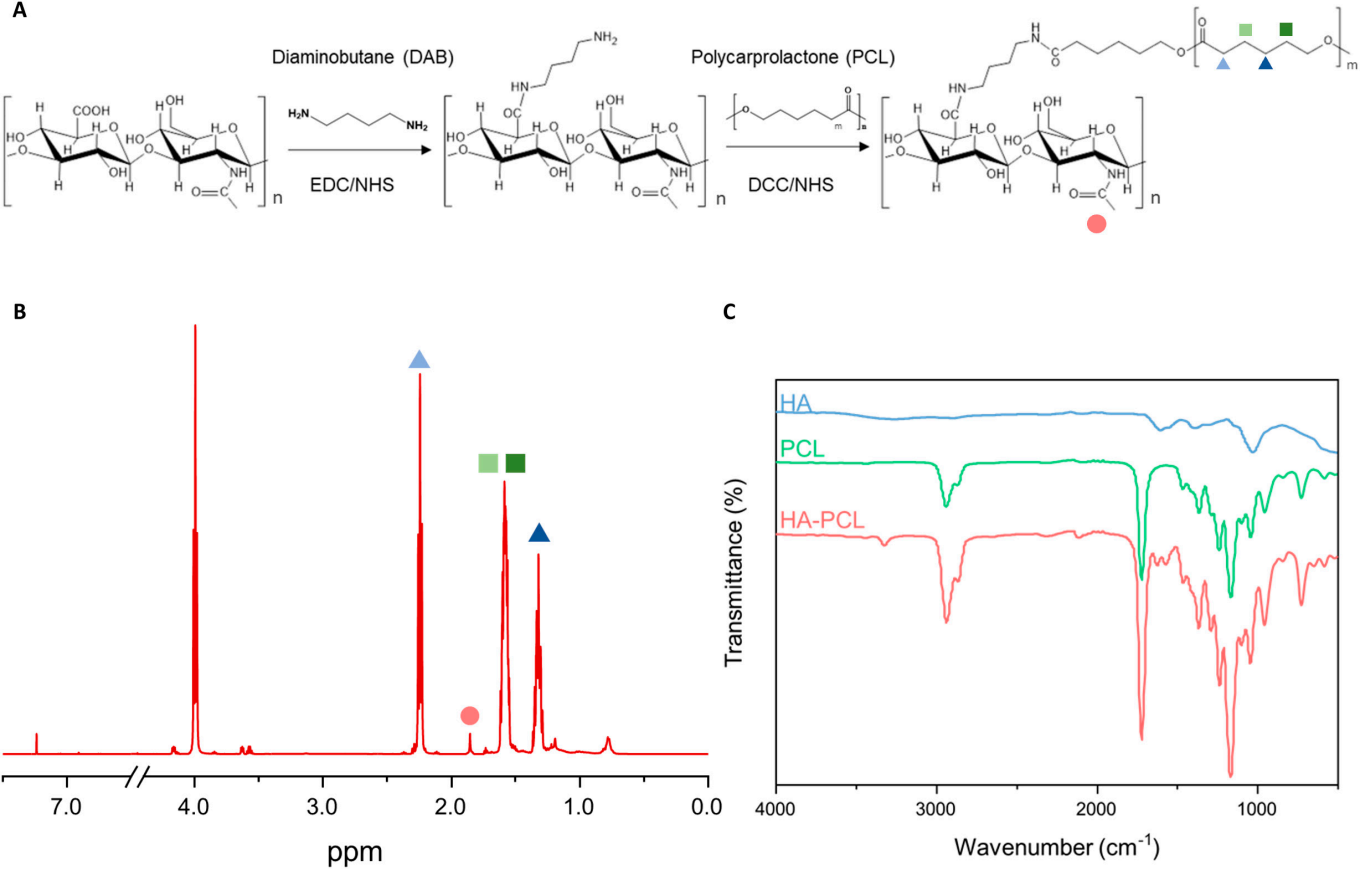
Preparation and characterization of HA-PCL conjugate. (A) Schematic illustration for the synthesis of the HA-PCL conjugate by a coupling reaction between HA-DAB and PCL. (B) ^1^H-NMR spectra of the HA-PCL conjugate in CDCl_3_. (C) FTIR spectra of pure HA, PCL, and the HA-PCL conjugate.

The synthesis of water-dispersible and transdermal deliverable red-to-blue upconverting HA-PCL/UC NPs was carried out as follows (Fig. 2A). Prior to this synthesis, DAB was grafted onto HA (HA-DAB) via EDC and NHS chemistry to introduce amine group into HA. Subsequently, the carboxyl groups of PCL were conjugated to the amine groups of HA-DAB via DCC and NHS. The successful synthesis of the HA-PCL conjugates was confirmed through ^1^H-NMR and Fourier transform infrared (FTIR) spectroscopy. As depicted in Fig. 2B, the characteristic peak of HA (-NHCOCH_3_) was observed at δ = 1.86 ppm, while the characteristic peak of PCL (-CH_2_COO-) was detected at δ = 2.25 ppm in the ^1^H-NMR spectrum. The substitution ratio of PCL to HA was approximately 5%, as confirmed by their respective characteristic peak areas. Further characterization of the HA-PCL conjugate was conducted using FTIR spectroscopy and compared with that of pure HA and PCL (Fig. 2C). The FTIR spectrum revealed the presence of typical HA bands, including the O–H stretching vibration (3,283 cm^−1^), C–O–C stretching vibration (1,044 cm^−1^), and the carbonyl group of the carboxylate (COO-) asymmetric stretching vibration (1,614 cm^−1^). In contrast, the representative peaks of PCL included CH_2_ asymmetric and symmetric stretching vibrations (1,240 and 1,170 cm^−1^ respectively), C=O stretching vibration (1,727 cm^−1^) in the carbonyl groups, and C–O–C asymmetric and symmetric vibrations (2,950 and 2,865 cm^−1^ respectively).

**Fig. 3.**
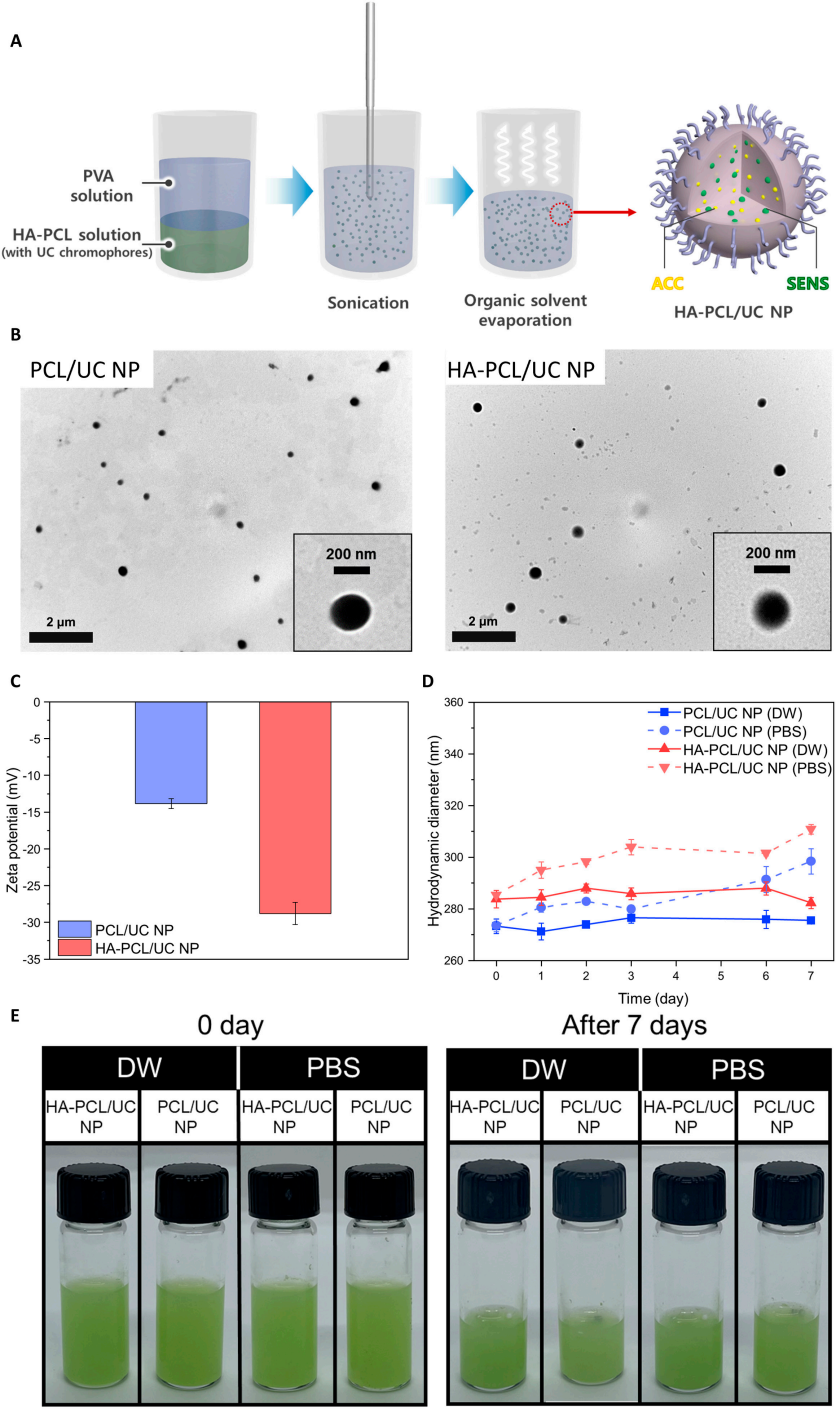
Preparation and characterization of HA-PCL/UC NPs. (A) O/W emulsion solvent evaporation method for the formation of HA-PCL/UC NPs. (B) TEM images of PCL/UC NPs and HA-PCL/UC NPs. (C) Zeta potential measurement of PCL/UC NPs and HA-PCL/UC NPs (mean ± SD; *n* = 3). (D) Seven-day particle stability evaluation of PCL/UC NP and HA-PCL/UC NP hydrodynamic sizes in DW and PBS (mean ± SD; *n* = 3). (E) Dispersion stability test in DW and PBS with a 10 mg ml^−1^ concentration after 0 and 7 d.

For the preparation of HA-PCL/UC NPs, we employed the O/W emulsion method using a tip homogenizer (Fig. 3A). To evaluate the enhanced particle stability, cellular uptake, and transdermal delivery efficiency attributed to HA, we also synthesized particles without HA conjugation (PCL/UC NPs) for comparison. TEM analysis revealed the formation of uniform spherical NPs, with sizes of 270 nm for PCL/UC NPs and 280 nm for HA-PCL/UC NPs, respectively (Fig. 3B). Hydrodynamic size analysis confirmed that the sizes of PCL/UC NPs and HA-PCL/UC NPs were 273.3 ± 2.81 nm (polydispersity index: 0.02822) and 283.7 ± 3.78 nm (polydispersity index 0.03722), respectively. Zeta potential measurements revealed negative charges of −13.87 ± 0.666 mV for PCL/UC NPs and −28.81 ± 1.505 mV for HA-PCL/UC NPs (Fig. 3C). Notably, HA-PCL/UC NPs exhibited a more pronounced negative charge, attributed to the carboxyl group of HA, which imparted strong electrostatic repulsion, preventing the aggregation of particle in water. Additionally, particle stability analysis was conducted by dispersing PCL/UC NPs and HA-PCL/UC NPs at a concentration of 10 mg ml^−1^ in DW and PBS for 7 d (Fig. 3D). Following the hydrodynamic size measurements, both PCL/UC NPs and HA-PCL/UC NPs showed slight alterations of approximately 1.46% and 0.8% in DW and approximately 9.1% and 8.9% in PBS, respectively. Visual inspection demonstrated no significant changes (Fig. 3E and F). These results indicate that the synthesized HA-PCL/UC NPs possess stable aqueous dispersibility and small size, which are crucial for effective delivery to cells or the skin, as supported by subsequent cell experiments and transdermal delivery efficiency evaluations.

### Optical properties of HA-PCL/UC NPs in aqueous phase

**Fig. 4.**
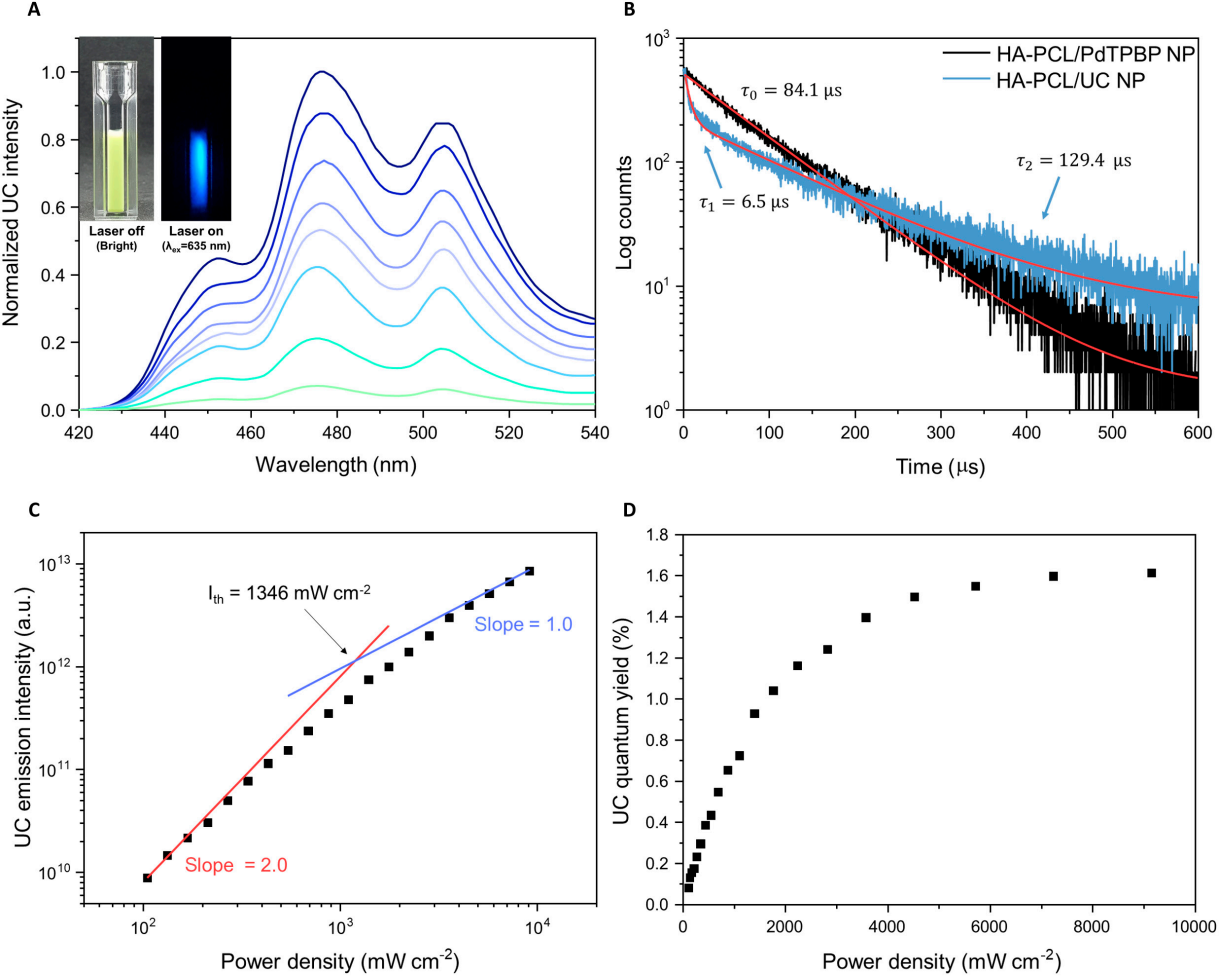
(A) UC emission spectrum of HA-PCL/UC NPs with increasing 635-nm laser excitation. (B) Phosphorescence decay profiles of HA-PCL/PdTPBP NPs and HA-PCL/UC NPs at 800 nm following excitation at 635 nm. (C) Integrated UC emission intensity and (D) UC quantum yields of the HA-PCL/UC NPs against the power density of 635-nm laser excitation. A 632-nm notch filter was used to remove the scattered excitation light.

As shown in Fig. 4, the optical properties of HA-PCL/UC NPs were characterized. To achieve red-to-blue UC, PdTPBP was chosen as the sensitizer and perylene was selected as the acceptor, and these chromophores were loaded into HA-PCL NPs. PdTPBP showed absorption bands centered at 440 nm (Soret band) and 635 nm (Q-band) and emitted phosphorescence centered at 800 nm (Fig. S1). On the other hand, perylene exhibited finger-like absorption band around 400 nm and emitted fluorescence at around 440 nm. PdTPBP and perylene have been well-known representative pairs of chromophores for the implementation of the red-to-blue TTA-UC by the energy transfer processes (Fig. S2) [32,33]. The detailed processes are as follows: (a) Initially, PdTPBP was excited to the singlet state (^1^S*) under 635-nm irradiation. (b) The triplet excited state of PdTPBP (^3^S*) was then generated via the ISC, which is enhanced by the heavy atom effect of PdTPBP. (c) Through Dexter energy transfer, including triplet–triplet energy transfer (TTET) and TTA, the energy of the triplet excited state of PdTPBP is transferred to the singlet excited state of perylene (^1^A*), finally resulting in upconverted delayed fluorescence. This phenomenon was confirmed by measuring the photoluminescence (PL) spectra under irradiation by a 635-nm laser (Fig. 4A). Unlike the PL spectra of perylene in deoxygenated dichloromethane measured under 270-nm irradiation, where the highest PL peak was located at ca. 440 nm, the highest UC emission peak was around 470 nm due to the self-absorption of the high concentration of perylene, which is inevitably required for effective TTA-UC. Further photochemical properties were obtained by analyzing the phosphorescence lifetime of HA-PCL/PdTPBP and HA-PCL/UC NPs (Fig. 4B). The phosphorescence of HA-PCL/PdTPBP NPs exhibited a monoexponentially decay with a lifetime of 84.1 μs (*τ_0_*). This lifetime was shorter than those reported in other media (e.g., deoxygenated THF and PCL film) [34,35], and we assumed that the nanoscale media were more affected by oxygen. In contrast, HA-PCL/UC NPs exhibited a biexponential decay with a short lifetime of 6.5 μs (*τ*_*1*_) and a long lifetime of 129.4 μs (*τ*_*2*_). These results were attributed to the TTET process and the thermodynamic equilibrium between the triplet excited states of the sensitizer and acceptor via back-and-forth energy transfer processes, respectively. The TTET efficiency calculated from these results was approximately 92% [*Φ*_*TTET*_ *= (1 − τ*_*1*_*/ τ*_*0*_*) × 100*], indicating efficient energy transfer from the sensitizer to the acceptor. The validity of these results was confirmed by analyzing the UC emission lifetime of the HA-PCL/UC NPs (Fig. S3A) according to a previous study. Meroni et al. [36] reported that *τ*_*2*_ and the triplet lifetime of perylene (*τ*_*T*_) are almost identical in the TTA-UC system employing PdTPBP and perylene as the sensitizer and the acceptor, respectively. To demonstrate this, τ_T_ was obtained by tail fitting of the UC emission decay at 470 nm according to the relationship [*I*_*UC*_*(t) ∝ exp(−t/τ*_*UC*_*) = exp(−2t/τ*_*T*_*)*, where *τ*_*UC*_ is the UC emission lifetime] [37] and it was determined to be 158.4 μs, which is almost coincident with *τ*_*2*_. The TTA-UC performance of HA-PCL/UC NPs was further evaluated through the analysis of its threshold intensity and UC quantum yield (Fig. 4C and D). In a double logarithmic plot of the integrated UC emission intensity against laser power density, the UC intensity of HA-PCL/UC NPs gradually transitioned from quadratic to linear dependence with increasing laser power density. This transition is based on the established kinetics of the relationship between the intensity of upconverted delayed fluorescence and the triplet excited state of the acceptor in the TTA-UC process. The quadratic-to-linear crossover point (i.e., threshold intensity, *I*_th_) represents the laser power density where TTA-UC occurs efficiently (~38.2% of its maximum UC quantum yield) [38], and *I*_th_ of the HA-PCL/UC NPs was 1,346 mW cm^−2^. The UC quantum yield of HA-PCL/UC NPs was calculated by relative method (details in Materials and Methods). According to the definition of quantum yield (the ratio of events to photons absorbed), the maximum quantum yield for UC is 50%, owing to its distinctive process in which 2 or more incident photons are transformed into a single upconverted photon. The UC quantum yield of HA-PCL/UC NPs was measured to be 1.6%, which represents a remarkable 78% increase compared with the highest UC quantum yield observed for polymeric NPs with the identical sensitizer and acceptor, which stood at 0.45% [39]. Furthermore, HA-PCL/UC NPs exhibited excellent photostability, retaining the UC intensity of the original value even under continuous irradiation for 2 h (Fig. S3B). The utilization of PCL as the core of UC NPs is significant due to its inherent physical strength and low oxygen permeability, which affords protection against oxygen quenching from the surrounding environment while facilitating chromophore diffusion [40]. In addition, the incorporation of HA is well recognized strategy for enhancing the solubility of water-insoluble nanomaterials for biomedical applications. Therefore, our HA-PCL/UC NPs, characterized by a hydrophobic PCL core enveloped by a hydrophilic HA shell, emerged as a judicious choice to serve as a stable light source in photomedicine and can pave the way for overcoming the limitations of existing issues in phototherapy and bioimaging.

### In vitro biocompatibility and UC assessment of HA-PCL/UC NPs

**Fig. 5.**
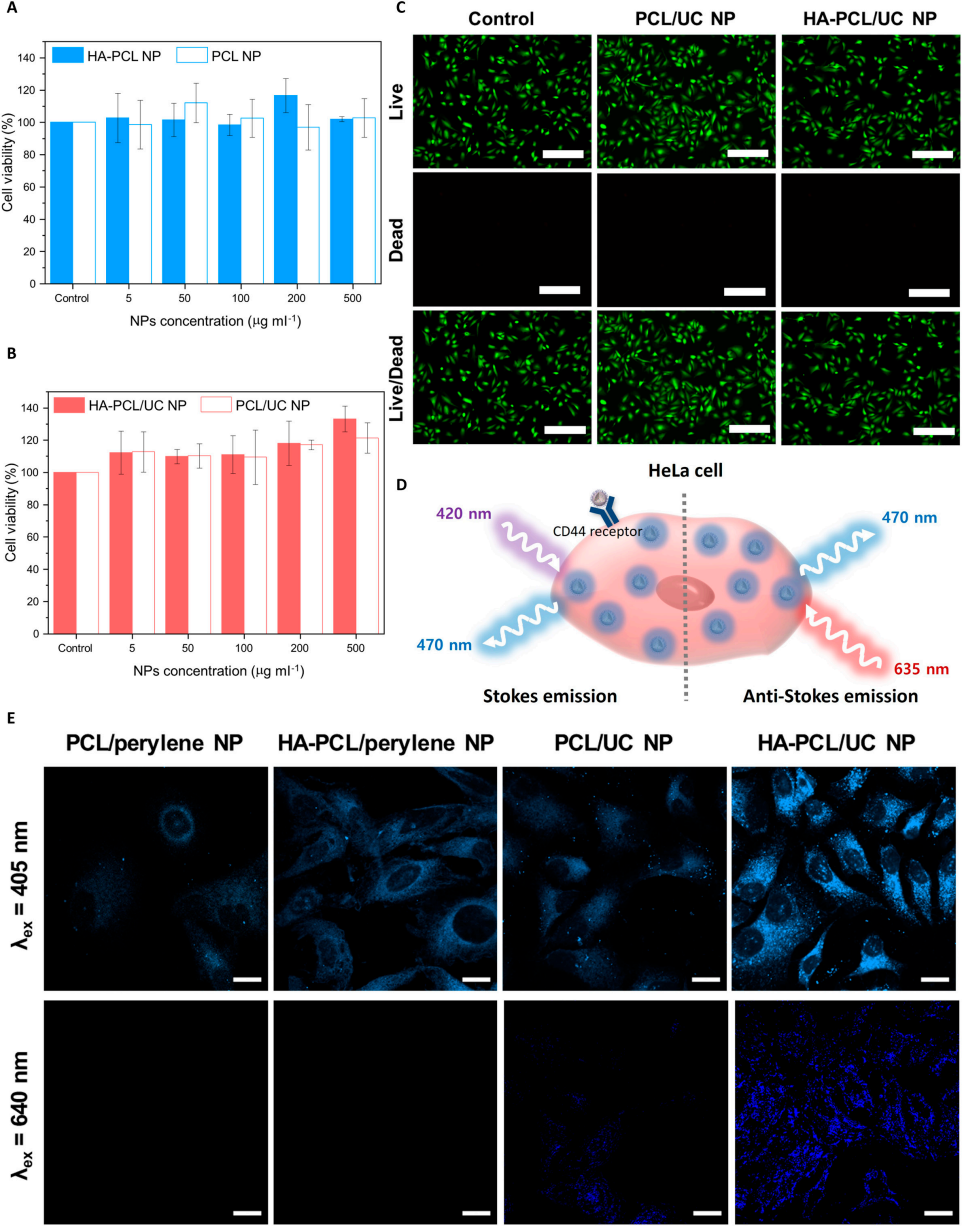
Cell viability assessment of (A) PCL NPs and HA-PCL NPs without TTA-UC chromophores and (B) with TTA-UC chromophores after incubation of HeLa cells with various concentrations of NPs (control, 5, 50, 100, 200, and 500 μg ml^−1^) for 24 h (mean ± SD; *n* = 3). (C) Fluorescence image of the Live/Dead assay after incubation of HeLa cells for 24 h. Green fluorescence means live cell, and red fluorescence means dead cell. Scale bar, 300 μm. (D) A diagrammatic representation of HeLa cell capable of emitting upconverted blue light through cellular uptake facilitated by the HA receptor. (E) CLSM images of the cellular uptake with different NPs (PCL/perylene and HA-PCL/perylene after incubation for 4 h in HeLa cells). Scale bar, 20 μm.

To investigate the biocompatibility of our photomedicine nanoplatform, we conducted cytotoxicity tests on both PCL/UC and HA-PCL/UC NPs using the human cervical cancer cell line (HeLa) and the mouse fibroblast cell line (L929). The cells were incubated with varying concentrations (ranging from 0 to 500 μg ml^−1^) of particles for 24 h. The CCK-8 assay revealed that both PCL/UC and HA-PCL/UC NPs exhibited no significant cytotoxicity, regardless of the presence of the hydrophobic UC chromophore (Fig. 5A and B and Fig. S4A and B). To further assess the potential cytotoxicity of the UC dyes, we conducted an additional experiment in which HeLa and L929 cells were cultured with HA-PCL/UC NPs for 72 h (Fig. S4C). Moreover, the Live/Dead assay further confirmed the excellent biocompatibility, as no red fluorescence signals were detected, indicating the absence of cell death in either cell type (Fig. 5C and Fig. S5).

In vitro imaging utilizing anti-Stokes emissions is a crucial biomedical application of UC NPs, owing to their exceptional photonic properties. These UC NPs disclose remarkable characteristics, including ultra-high signal-to-noise ratios and minimal autofluorescence, making them ideal for precise and sensitive imaging in biological systems [41,42] To validate the practical potential of HA-PCL/UC NPs, we performed cellular uptake experiments by incubating HeLa cells with the NPs and then visualizing the results using CLSM, which is performed in the 450- to 550-nm channel. In the optical analysis, perylene emitted blue light (Stokes emission) upon irradiation with a 405-nm laser and the combination of PdTPBP and perylene also exhibited blue light emission (anti-Stokes emission) under 640-nm laser irradiation, representing the photon UC process (Fig. 5D). The experiment involved 4 distinct groups: PCL/perylene, HA-PCL/perylene, PCL/UC, and HA-PCL/UC NPs, with the goal of clearly differentiating and visualizing the emissions of Stokes and anti-Stokes from the cells, enabling a comprehensive analysis of the cellular uptake and localization of the NPs (Fig. 5E). Upon exposure to a 405-nm laser, all groups displayed blue Stokes emission from perylene. However, the HA-PCL/perylene NPs and HA-PCL/UC NP groups exhibited particularly brighter fluorescence. Conversely, when irradiated with a 640-nm laser, only the HA-PCL/UC NP group showed evident blue UC emission. This observation suggests that HA-mediated endocytosis occurred, likely facilitated by the HA receptor, CD44, which is known to be overexpressed on the HeLa cell surface [43]. Consequently, the HA-PCL/UC NPs demonstrated the highest cellular uptake efficiency, indicating successful cellular internalization. These results highlight the promising potential of HA-PCL/UC NPs for advanced cellular imaging and underscore their applicability in photomedicine, particularly in the areas of targeted imaging and diagnostics within biological systems.

### Penetration and UC imaging of HA-PCL/UC NPs in porcine skin

**Fig. 6.**
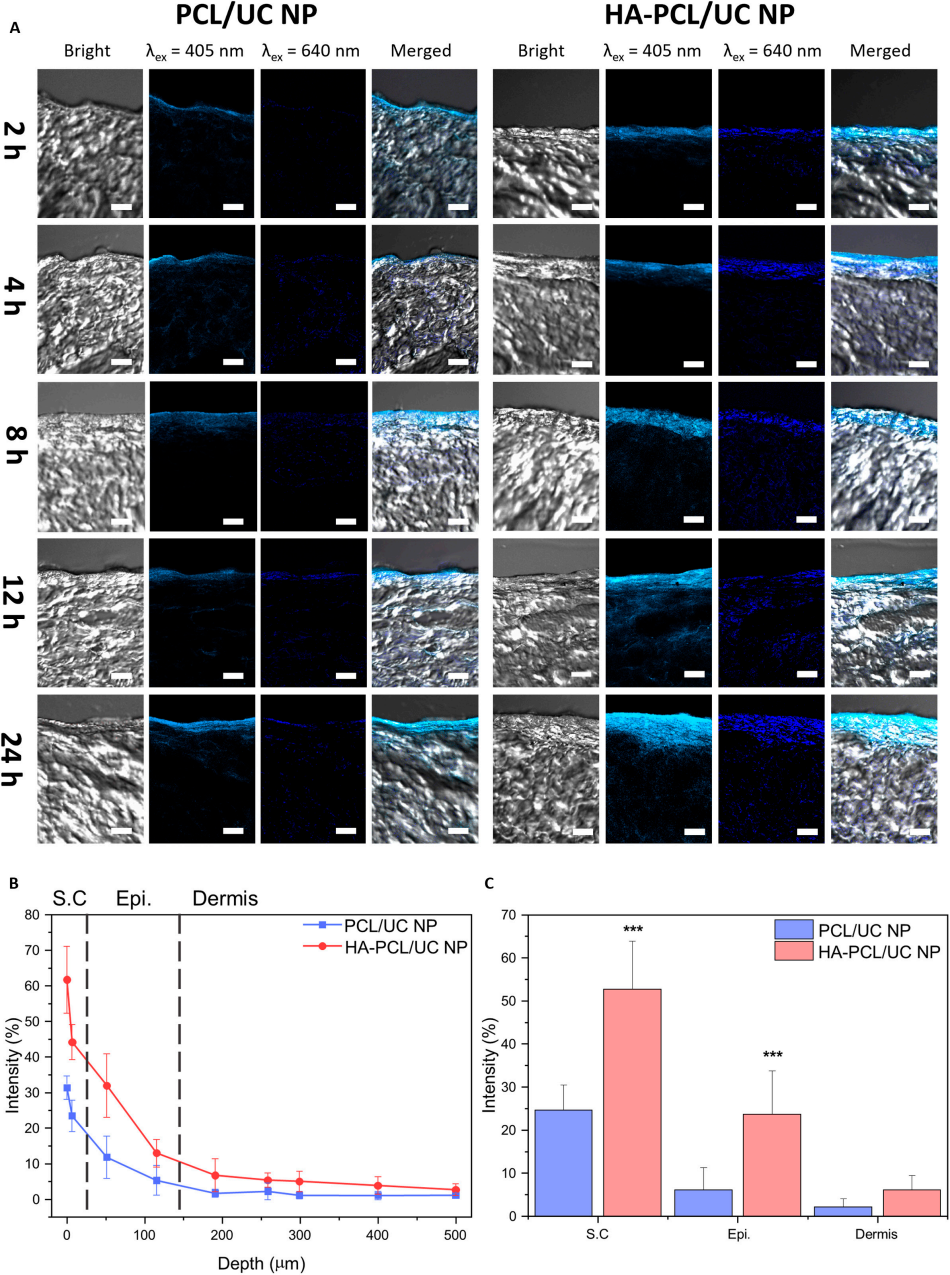
(A) CLSM images of histological cryosectioned porcine skin tissue after administration of PCL/UC and HA-PCL/UC NPs. The 405- and 640-nm lasers (maximum laser intensity: 5 mW) were used to obtain Stokes and anti-Stokes emission, respectively. Scale bar, 100 μm. (B) Fluorescence intensities under 640-nm laser irradiation of PCL/UC and HA-PCL/UC NPs at 12 h. (C) Quantification of upconverted fluorescence in the stratum corneum (S.C), epidermis (Epi.), and dermis layers (mean ± SD; *n* = 7; ***P < 0.001).

In our study, we explored the potential utility of HA-PCL/UC NPs as promising vehicles for TDDS. To assess skin penetration capabilities, we conducted experiments using porcine skin, which closely resembles human skin and has been widely used in previous studies [44]. Fig. 6 presents images of cryosectioned porcine skin after applying a solution of PCL/UC and HA-PCL/UC NPs dispersed in PBS (500 μg ml^−1^), captured at different time points (2, 4, 8, 12, and 24 h) after administration, revealing various fluorescence intensities across distinct skin layers. Under 405-nm laser irradiation, both groups displayed clear blue fluorescence attributed to perylene after 24 h (Fig. 6A and Fig. S6). Moreover, upon 640-nm laser irradiation, HA-PCL/UC NPs exhibited conspicuous and intense blue fluorescence attributable to photon UC. As shown in Fig. 6B, the anti-Stokes fluorescence profile as a function of depth originating from HA-PCL/UC NPs demonstrated their successful infiltration deep into the dermis layer, indicating their ability to effectively reach the desired target site within the skin after 12 h. Furthermore, a noteworthy UC emission intensity manifested in the stratum corneum and epidermis layers of the porcine skin in comparison to PCL/UC NPs. The considerably low efficiency of PCL/UC NPs in traversing the thick skin layer, namely, the stratum corneum, represents a chronic limitation of existing TDDS. Importantly, the incorporation of HA, a principal constituent of the skin, into the HA-PCL/UC NPs enabled them to penetrate the stratum corneum of the skin more efficiently, allowing deep penetration into the dermis (Fig. 6C). Notably, HA has been a prominent choice in numerous studies including therapeutics [45], plastic surgery [46], and cosmetic formulations [47,48]. These findings align with the pivotal role played by HA in facilitating superior permeability, owing to its inherent amphiphilic nature. The remarkable hydration capacity of HA serves to disrupt the densely packed structure of corneocytes, thereby enabling it to permeate through intercellular gaps. Furthermore, its amphiphilic nature plays an essential role by facilitating interactions with both hydrophilic and hydrophobic components of the skin, thereby promoting penetration through the intercellular lipids [49]. Considering the demonstrated proficiency in TDDS along with the previously evidenced in vitro UC imaging capability of HA-PCL/UC NPs, they present promising potential as a novel platform for biomedical applications. Moreover, there exists a promising avenue for the development of a system that may synergistically combine noninvasive light energy irradiation attributes inherent to optical medicine. This holds the potential to unveil captivating prospects in photomedicine.

## Discussion

We have developed red-to-blue upconverting HA-PCL/UC NPs composed of PdTPBP and perylene, which successfully overcome several obstacles, including the low drug delivery efficiency of TDDS, the low biopermeability of photomedicine, and the quenching effect induced by oxygen molecules in TTA-UC. The fabricated HA-PCL/UC NPs were spherical in shape and monodisperse in size, ensuring stable aqueous dispersibility. The conjugation of HA notably improved the endocytosis efficiency, particularly in HeLa cells, due to the overexpression of CD44 receptors. This enhancement underscores their potential as a versatile targeted in vitro imaging platform using anti-Stokes emissions and enhances their transdermal delivery efficiency. Because of the distinctive properties of HA, including its remarkable hydration capacity, which can disrupt corneocytes, and its amphiphilic nature, which facilitates interactions with both hydrophilic and hydrophobic components of the skin, it significantly enhances skin permeability. A pivotal aspect of HA-PCL/UC NPs is the embedding of UC chromophores within the PCL core. This hydrophobic core, characterized by low oxygen permeability, protects the NPs from oxygen molecules and facilitates energy transfer. Consequently, this structure ensured a superior UC quantum yield (1.6%) in the aqueous phase, representing a 78% improvement over the previous polymeric UC NPs with the same chromophores.

The synergy between the inherent optical properties of UC and noninvasive TDDS holds the potential to yield highly effective therapeutic outcomes deep within the layers of dermal tissue. Moreover, this approach can impart multifunctionality through light-induced triggers, by utilizing noninvasive techniques that convert long-wavelength light into short-wavelength light. This area of research has only been explored by a limited number of researchers. In our previous study, we demonstrated that integrating cryotherapy with photodynamic therapy using cold-responsive Ln UC nanoplatforms coated with HA synergistically boosted skin cancer treatment efficacy [50]. We also introduced an innovative noteworthy strategy employing HA-based inorganic UC NPs for achieving noninvasive photochemical tissue bonding using NIR light [13]. Prieto et al. [51] demonstrated that PLGA-PEG NPs, coencapsulating inorganic UC NPs and protoporphyrin IX, can emit UV and visible light, significantly enhancing skin penetration and promoting the generation of reactive oxygen species upon 808-nm light irradiation. Nonetheless, it is worth noting that all these instances rely on inorganic UC materials. Consequently, significant challenges remain in developing innovative materials that can address the limitations associated with these inorganic UC materials.

In summary, the newly developed HA-PCL/UC NPs represent the first application of TTA-UC-based phototherapy for noninvasive TDDS. Their potential to overcome the limitations of short-wavelength light is augmented by enhanced skin penetration, exceptional particle stability, and superior optical properties in aqueous media, thereby paving the way for novel advancements in photomedicine applications. These NPs have the potential to revolutionize deep tissue bioimaging, photo-triggered drug delivery, antibacterial therapy, and photodynamic therapy, offering targeted and effective therapeutic interventions in the field of photomedicine. Further research and optimization of our novel UC nanoplatform hold the key to unlocking its full potential and can lead to novel and impactful advancements in the field of photomedicine combined with dermatology.

## Data Availability

The data supporting the findings of this study are available from the corresponding author upon reasonable request.
