# Supplementary material for: Water-Dispersible and Biocompatible Polymer-Based Organic Upconversion Nanoparticles for Transdermal Delivery
Source: Biomater Res. 2024 Nov 19;28:0106. doi: 10.34133/bmr.0106 (PMC11574081; doi:10.34133/bmr.0106)
Supplement: Supplementary 1 — Figs. S1 to S6 [file bmr.0106.f1.docx]

Supplementary Materials

**Water-dispersible and Biocompatible Polymer-based Organic Upconversion Nanoparticles for Transdermal Delivery**

**Organic Upconversion Nanoparticles for Transdermal Delivery**

Hye Eun Choi^1,^ ^†^, Jeong-Min Park^2,^ ^†^, Woo Yeup Jeong^1^, Su Bin Lee^1^, Jae-Hyuk Kim^2, *^, Ki Su Kim^1,3, *^

^1^ School of Chemical Engineering and Institute for Advanced Organic Materials, Pusan National University, Busan 46241, Republic of Korea

^2^ Department of Civil and Environmental Engineering, Pusan National University, Busan 46241, Republic of Korea

^3^ Department of Organic Materials Science and Engineering, Pusan National University, Busan 46241, Republic of Korea

^*^ Address correspondence to: kisukim@pusan.ac.kr (K.S.K.), jaehyuk.kim@pusan.ac.kr (J.-H. K.)

† Theses authors contributed equally to this work.


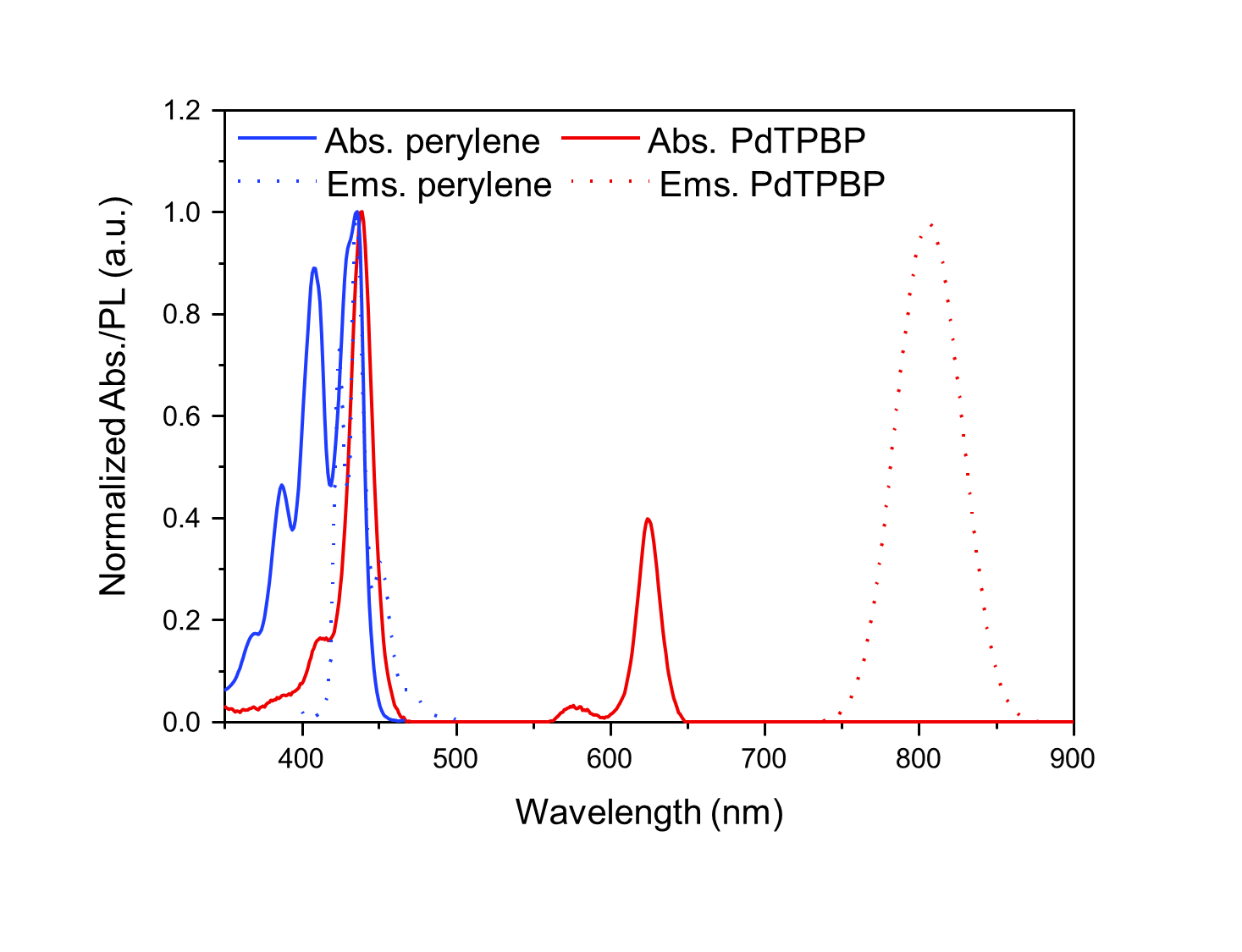


**Fig. S1.** Normalized absorption (solid) and emission (dashed) spectra of PdTPBP (red) and perylene (blue) in dichloromethane**.**


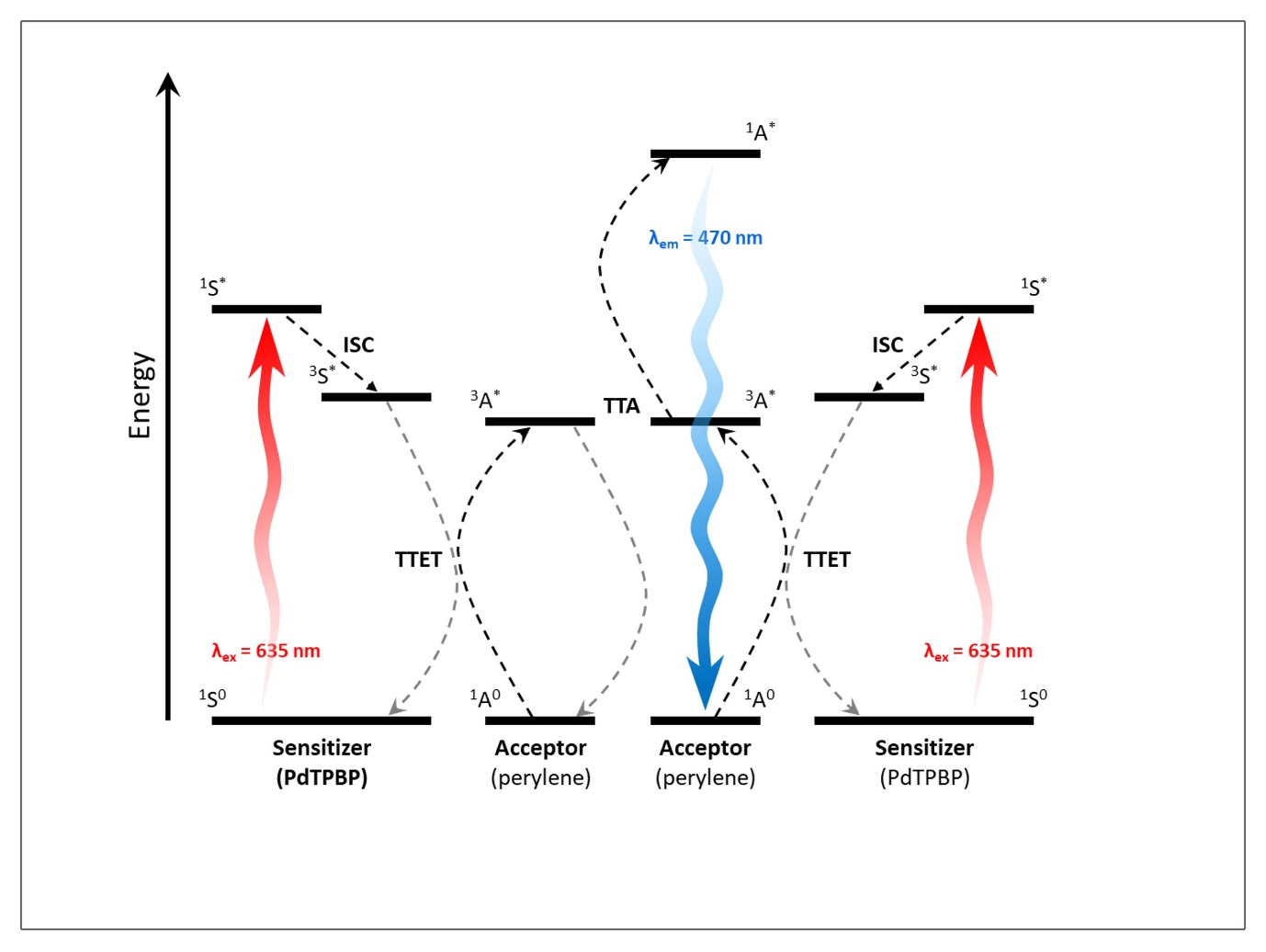


**Fig. S2.** A Jablonski diagram describing triplet-triplet annhilation upconversion (TTA-UC) proce.


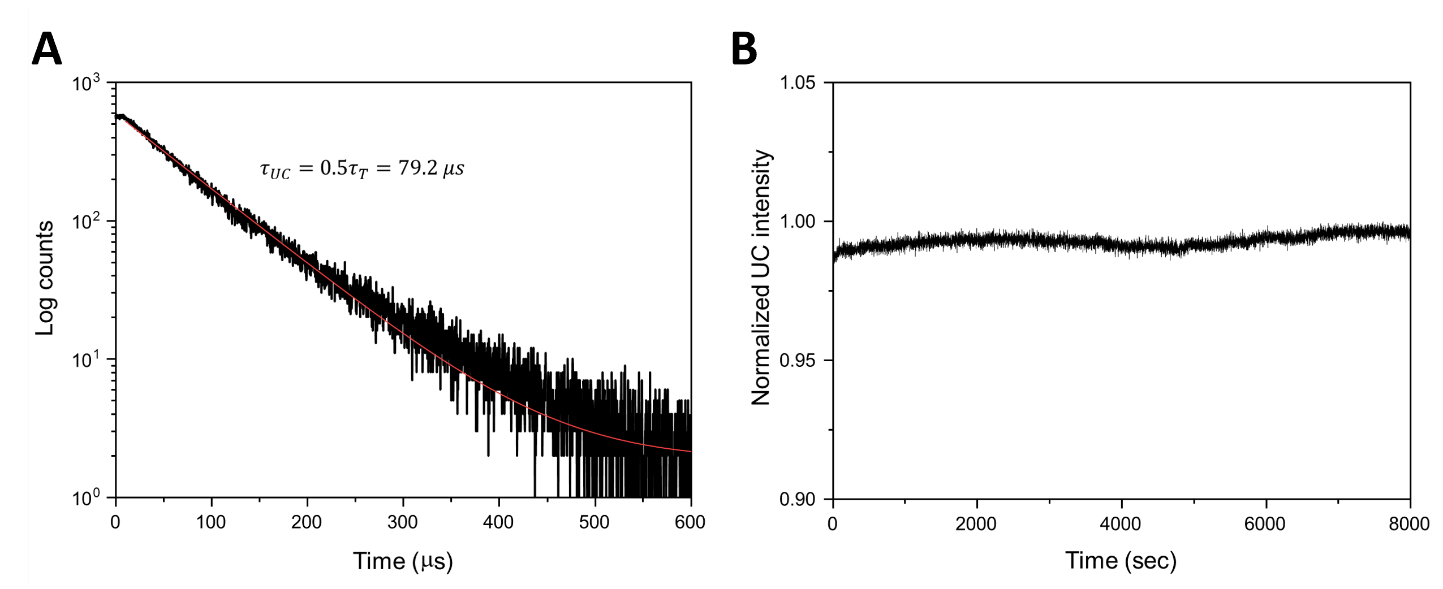


Fig. S3. (A) UC emission decay (at 470 nm) of HA-PCL/UC NPs under excitation at 635 nm. (B) UC emission stability (at 470 nm) of HA-PCL/UC NPs under continuous excitation at 635 nm (103 mW cm^-2^).


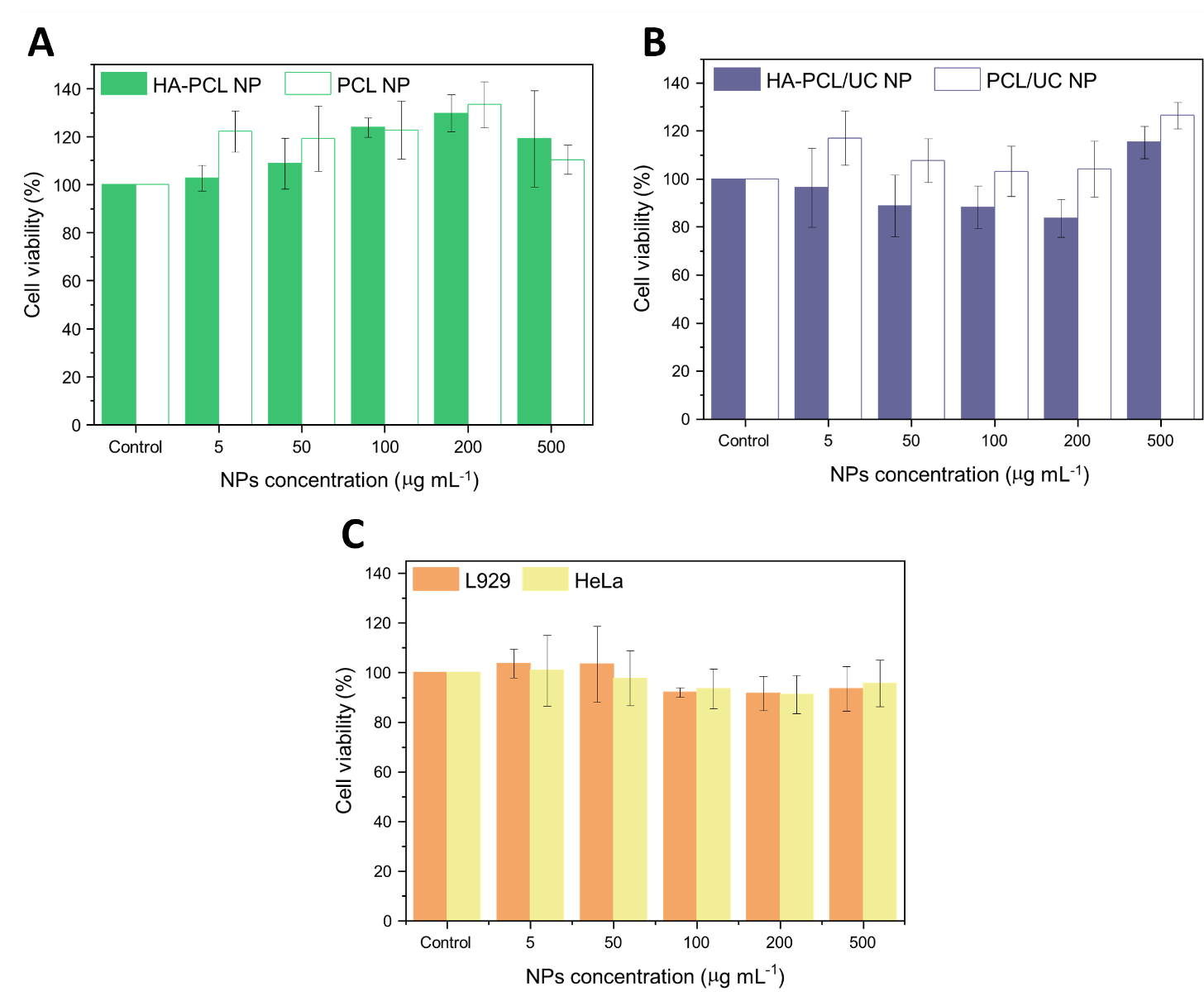


**Fig. S4.** Cell viability assessment of (A) PCL NPs and HA-PCL NPs without TTA-UC chromophores and (B**)** with TTA-UC chromophores after incubation of L929 cells with various concentrations of nanoparticles for 24 h and (C) HA-PCL/UC NPs after incubation of L929 and HeLa cells with various concentrations of nanoparticles for 72 h (mean ± SD; n=3).

**
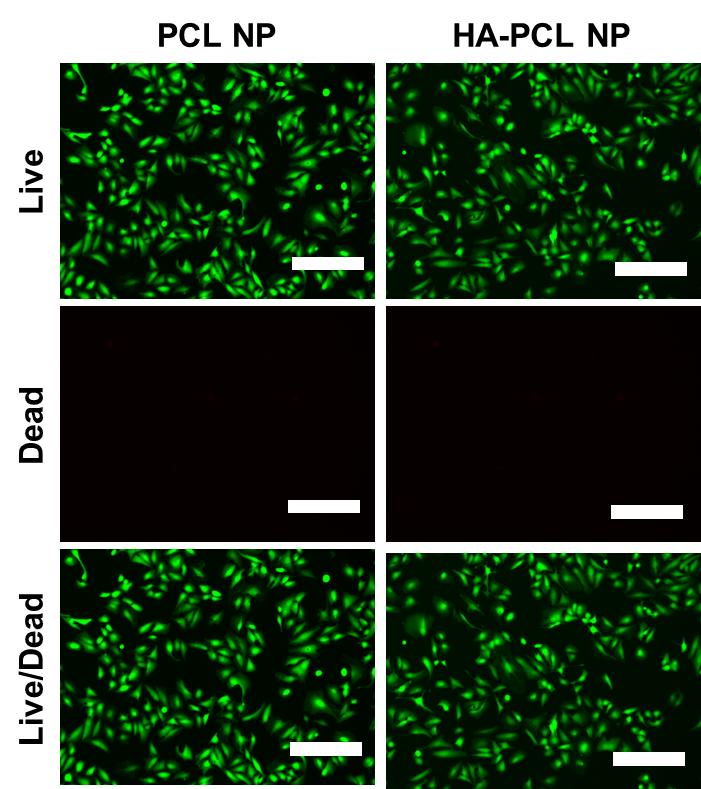
**

**Fig. S5.** Live/Dead assay of PCL/UC and HA-PCL/UC NPs after incubation for 4 h in L929 cells. Scale bar 300 μm


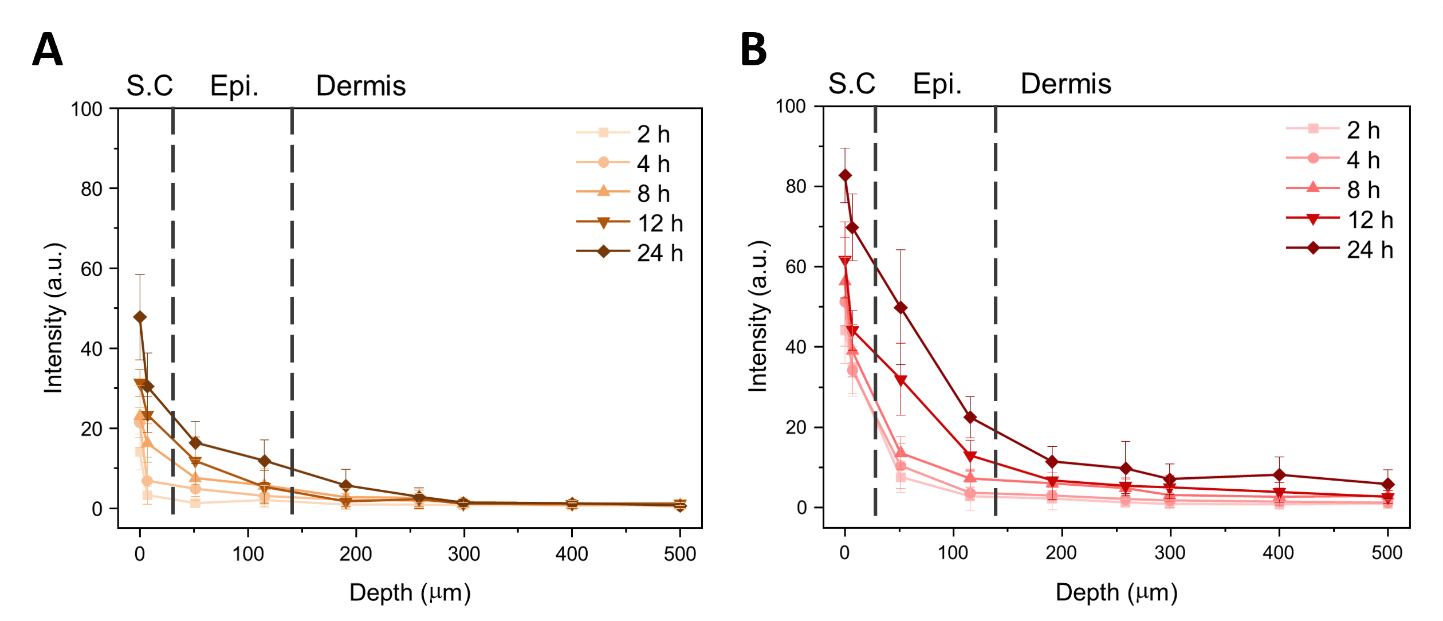


**Fig. S6.** Fluorescence intensity graph under 640 nm laser irradiation after administration (A) PCL/UC NPs and (B) HA-PCL/UC NPs (mean ± SD; n=7)
